# Coparenting and children’s socio-emotional ability: the mediating role of parental stimulation

**DOI:** 10.3389/fpsyg.2025.1505267

**Published:** 2025-09-04

**Authors:** Xuena Wang, Zhongqin Huang, Jun Zhong, Suqing Wang, Liping Yu

**Affiliations:** ^1^Zhongnan Hospital, Wuhan University, Wuhan, China; ^2^School of Nursing, Wuhan University, Wuhan, China; ^3^Zigong Fourth People’s Hospital, Zigong, China; ^4^Center for Nurturing Care Research, School of Nursing, Wuhan University, Wuhan, China

**Keywords:** coparenting, parental stimulation, children’s social–emotional ability, nurturing care, mediating role

## Abstract

**Background:**

Previous research has inadequately examined the interconnections among coparenting, parental stimulation, and children’s social–emotional ability within the context of Chinese culture.

**Aim:**

Consequently, this study, guided by the coparenting ecological model framework, aims to investigate these relationships and to determine whether parental stimulation serves as a mediating factor.

**Methods:**

In this study, 330 mothers of children aged 12–36 months were selected in Community Health Service Center from April to October 2021 in Wuhan. The general information questionnaire, the Chinese version of Brief Coparenting Relationship Scale, the Infant and Toddler Social and Emotional Assessment, and the Family Care Indicators were distributed. AMOS 21.0 statistical software was used for mediating effect analysis.

**Results:**

In terms of social emotion, 10.3% of children had abnormal social emotion. Coparenting, parental stimulation, and children’s social–emotional ability pairwise showed a positive correlation. Parental stimulation mediated the association between coparenting and social–emotional ability (*β* = 0.011, bootstrap 95% *CI* = 0.006, 0.018). Paternal stimulation and maternal stimulation played a chain mediating role in the relationship between coparenting and social–emotional ability (*β* = 0.004, bootstrap 95% *CI* = 0.002, 0.008).

**Conclusion:**

Children’s social–emotional ability may be enhanced through interventions that guide and enhance coparenting and parental stimulation.

## Introduction

The first 1,000 days of an individual’s life is the period of the strongest brain plasticity. Neurons establish new connections at an astonishing rate of 700–1,000 per second. During this period, the brain experienced rapid development (Fox et al., 2010). The development of children during this period will affect their future physical development, mental health, educational trajectory, salary level, etc. (Walker et al., 2011). According to statistics, more than 250 million children under the age of 5 have not reached their developmental potential in low-and middle-income countries (Black et al., 2017). High-quality nurturing care is conducive to children’s growth and development, can promote children’s early development, and has an impact on children’s life (Black et al., 2017). In recent years, the importance of nurturing care for early childhood development has been recognized. In 2018, WHO and other international organizations jointly released the Nurturing Care Framework (NCF), including health, nutrition, security and safety, responsive caregiving, and early learning (World Health Organization, United Nations Children’s Fund, World Bank Group, 2018).

Early childhood development refers to the full development of children’s cognitive, motor, social–emotional, and physical potential between the ages of 0 and 8 (World Health Organization, 2020). Compared with other developmental aspects, social emotion is an easily overlooked part (Malmberg et al., 2016; Rempel et al., 2017). As an important part of the psychological development of children, the early social–emotional ability can effectively predict their problem-solving ability and interpersonal relationship (Wang et al., 2019; Williams and Lerner, 2019), and it is also closely related to their future emotional behavior problems and academic performance, and can even predict their future personality development (Wang et al., 2019; Williams and Lerner, 2019). There are large individual differences in children’s social–emotional development. Apart from the influence of children’s gender and temperament type, it is also largely influenced by parents’ nurturing care and early family environment (Black et al., 2017).

For infants and young children, the family is the first environment for them to receive nurturing and social development after birth, and the family environment is often considered to be one of the most important factors affecting children’s development. The family environment usually includes the natural environment and the psychosocial environment. The natural environment includes family living conditions, socioeconomic status, and the surrounding environment of the residence, etc. (McLeroy et al., 1988). Family nurturing environment refers to the psychosocial environment of the family, including parenting attitudes of parents, stimulating learning environment (such as books, toys, etc.), parents and children participating in stimulating activities (such as reading, singing, storytelling, and letting children have different experiences) and parents can identify children’s needs promptly and give appropriate and warm responses promptly (Black et al., 2017). A good family nurturing environment contributes to the early cognitive, language, social–emotional, and motor development of children (Black et al., 2017). Therefore, it is necessary to pay attention to the family nurturing environment in early childhood, and this paper mainly focuses on the stimulation of father and mother, key factors in promoting the early development of children.

Family system theory shows that in the whole family system, the participation of other caregivers other than the mother (such as father) expands the mother–child relationship into a tripartite relationship. When the three parties establish a good relationship and bring the system into a balanced state, children can benefit from it (Marsanic and Kusmic, 2013). Coparenting describes how parents coordinate their shared responsibility in childrearing by supporting or undermining one another’s parenting efforts (Tissot et al., 2017). Coparenting involves both fathers and mothers in our study. In China, men’s roles in the family have changed due to demographic, socioeconomic, and cultural shifts in recent years. For example, mothers not only take on family care responsibilities but also participate in some social work, leading fathers and mothers to share family responsibilities, including parenting and supporting the healthy development of their children. In the research on nurturing care and early childhood development, more attention is paid to the role of mothers, such as exploring the relationship between mothers’ responsive care and children’s social emotion and growth (Scherer et al., 2019). Compared with mothers, less research has focused on the role of fathers in parenting, but fathers play an important role in parenting. Studies have shown that (Wang et al., 2020) better paternal care and interaction can significantly reduce the risk of developmental delay in children within 2 years of age.

The ecological model of coparenting was proposed by Feinberg (2003). This model describes the impact of family system on coparenting from three levels of individual, family, and extra-family factors, as well as the possible mediating or moderating relationships between coparenting and child development outcomes. The quality of coparenting directly affects parenting and child development, and indirectly affects child development through parenting. Positive coparenting may create a family environment with a lower stress level for children, which will promote parenting adjustment and parent–child interaction, thus affecting children’s mental health. Previous studies have explored the relationship between coparenting and parental stimulation. Parents who support each other in coparenting show more responsiveness and interaction with their children (Zemp et al., 2018). In addition, previous studies have shown that good parental stimulation is beneficial to the early development of infants and young children. For example, a study in the United States (Cprek et al., 2015) showed that interactive activities such as reading, storytelling, singing, and eating with family members can reduce the risk of children’s developmental and behavioral delays. Besides, previous studies have also explored the relationship between coparenting and early child development, and the results show that the higher the quality of coparenting, the fewer children’s behavioral problems and the better children’s adaptation (Jacobvitz et al., 2022; Murphy et al., 2016; Teubert and Pinquart, 2010).

Although previous studies have shown that coparenting, parental stimulation, and social–emotional ability are correlated with each other (Zemp et al., 2018; Cprek et al., 2015; Murphy et al., 2016), few studies have explored the mediating role of parental stimulation in the middle, and limited studies have explored the relationship between the three variables in the context of Chinese culture. In the Chinese cultural context, harmonious family relations are emphasized. It is very important that parents cooperate with each other in parenting (Lau and Power, 2019). In addition, with the development of social culture, women are more and more involved in work, and fathers are gradually involved in parenting (Liu et al., 2016). However, there are few studies on Chinese fathers’ involvement in parenting. Therefore, under the guidance of the coparenting ecological model framework, this paper has two objectives. First, it aims to explore the relationship between coparenting, parental stimulation, and children’s social–emotional development in the context of Chinese culture; second, to explore whether parental stimulation (including paternal stimulation and maternal stimulation) plays a mediating role between the two variables. We hypothesized that paternal stimulation and maternal stimulation mediate the relationship between coparenting and children’s social–emotional ability.

## Methods

### Study design and participants

This study was approved by the Ethics Committee of Wuhan University School of Medicine. Convenience sampling was used in the child health department of three Community Health Service Centers in different regions of Wuhan from April to October 2021. Sample size was estimated as 280 by setting *α* as 0.05, 1-*β* as 0.95, and effect size as 0.25. The recruited participants should be ranged from 329 to 350 considering a loss to follow-up rate of 15–20%.

The parents were informed of the purpose and significance of the study while they were waiting for treatment. Three Hundred Sixty-nine mothers gave their informed consent to participate in the study and receive the questionnaires, and 330 met the inclusion and exclusion criteria. Inclusion criteria for participants: (1) mothers of young children aged 12–36 months; (2) being the child’s caregiver (living with the child); (3) being able to understand and complete the questionnaire. Exclusion criteria: (1) mothers with serious physical and mental illness, (2) toddlers with congenital malformations, acute and chronic diseases.

### Measures

#### Sociodemographic questionnaire

Based on reviewing the literature, the sociodemographic questionnaire, included both children’s (sex, age, birth weight, and gestational weeks, etc.) and parents’ data (parents’ age, education level, marital status, economic status, place of residence, number of children in the family, children’s main caregiver, etc.).

#### Brief Coparenting Relationship Scale (Brief-CRS)

The Brief-CRS was developed by Feinberg et al. (2012) in 2012 to assess the quality of coparenting, and adapted by Chinese researchers in 2017 with good reliability and validity. The scale contains 5 dimensions: mutual recognition (3 items), coparenting support (4 items), coparenting undermining (2 items), conflict (3 items), and division of labor (2 items). Items are scored on a 7-point Likert scale, with 0 representing “not at all consistent” and 6 representing “completely consistent.” Among them, the dimensions of mutual damage and conflict are reversed scores. The total score ranges from 0 to 84, with higher scores indicating higher perceptions of coparenting. In this paper, coparenting is reported by mothers, with higher scores indicating that women perceive more support from their spouses and that fathers perform better in coparenting. In this study, Cronbach’s *α* coefficient was 0.83.

#### Family Care Indicators (FCIs)

Parents’ stimulation was measured using an adapted version of the FCI tool which was developed by groups of experts organized by the UNICEF (Hamadani et al., 2010). Previous studies revised FCI into Chinese to adapt to the Chinese language and environment (Wang, 2019). The scale has 19 items and 5 dimensions, including the varieties of play materials, the sources of play materials, the play activities within 3 days, the number of magazines and newspapers in the home. This study chose the dimension of play activities to measure parental stimulation. The items include: reading books or picture books, telling stories, singing nursery rhymes, playing games outdoors, playing games with toys, and naming, counting and drawing. Ask the father and mother how often they played various interactive games with their children in the past week, “none” scored 0 points, “1–3 days/week” scored 1 point, “4–6 days/week” scored 2 points, “Almost every day” is scored 3 points, and the score ranges from 0 to 18 points. The higher the score, the better the parents’ stimulation to the child. The Cronbach’s *α* coefficient in this study were 0.89 and 0.92, respectively.

#### Infant and Toddler Social and Emotional Assessment (ITSEA)

The scale was compiled by the scholar (Briggs-G, 1998), and parents evaluate the social–emotional behavior of infants and young children. The scale Brief-CRS was translated and adapted for use in China in 2008. The revised scale has 145 items and includes four domains, namely internalizing, externalizing, regulatory, and competence domains. This research mainly focuses on the social–emotional ability of children and selects the field of ability in this scale. There are 35 items in this field, including 6 dimensions, namely compliance, imitation/play, attention, empathy, mastery motivation, and prosocial peer relations. Items are scored on a 3-point Likert scale: “0” means “disagreeable,” “1” means “partially consistent,” and “2” means “very consistent.” The higher the total score, the higher the children’s social–emotional ability. Convert the average score to the T score with a mean of 50 and a standard deviation of 10. If the *T* score is <37, it is a positive sign of delayed social–emotional development. The scale has good reliability and validity. In this study, Cronbach’s α coefficient was 0.93.

### Data analysis

Categorical data were expressed as frequency and percentage or proportion. Measurement data were expressed as mean and standard deviation (normal distribution) or as median and interquartile ranges (non-normal distribution). Correlation analyses were performed among parenting, parental stimulation, and children’s social–emotional ability. Hierarchical regression analysis was used to explore the influencing factors of social–emotional ability by SPSS 21.0. The mediating role of parental stimulation between coparenting and children’s social–emotional ability was analyzed by AMOS 21.0. *p* < 0.05 was considered statistically significant.

## Results

### Sociodemographic characteristics

A total of 369 questionnaires were issued, 330 valid and 39 invalid. Among the invalid questionnaires, 11 were incomplete, and 28 questionnaires were obviously wrong and consistent item options, with an effective rate of 89.4%. Table 1 describes the sociodemographic characteristics. Among them, 53.9% were boys; the age distribution of children was 34.8% from 12 to 18 months old; the main caregivers of young children are grandparents, accounting for 41.8%; 67.0% of families with only one child; the proportion of low-weight infants was 4.2%; the proportion of premature infants was 5.5%.

**Table 1:** Association between demographic characteristics and children’s social emotional ability (*N* = 330).

Variables	Classification	*N*(%)	*x– ± S*	*t/F*	*P*
Child					
Child’s gender	Boy	178(53.9)	46.82 ± 12.94	0.68	0.41
	Girl	152(46.1)	47.91 ± 10.88		
Child’s age (months)	12–24	207(62.7)	44.00 ± 11.97	48.72	<0.001
	24–36	123(37.3)	52.93 ± 9.89		
Number of children	One	221(67.0)	47.56 ± 11.86	0.26	0.611
	Two or more	109(33.0)	46.84 ± 12.39		
Feeding method	Breastfeeding	187(56.7)	48.57 ± 11.21	10.21	<0.001
	Artificial feeding	42(12.7)	39.71 ± 14.02		
	Mixed feeding	101(30.6)	48.19 ± 11.55		
Delivery way	Natural birth	168(50.9)	48.59 ± 11.60	3.82	0.052
	Cesarean delivery	162(49.1)	46.01 ± 12.36		
Low birth weight	Yes	14(4.2)	40.14 ± 10.41	5.28	0.022
	No	316(95.8)	47.64 ± 12.01		
Preterm birth	Yes	18(5.5)	46.44 ± 14.07	0.10	0.750
	No	312(94.5)	47.38 ± 11.92		
Parents					
Monthly family income (RMB)	<7,000	66(20.0)	43.13 ± 13.47	3.89	0.009
	7,000–10,000	101(30.6)	47.30 ± 11.42		
	10,000–20,000	103(31.2)	49.18 ± 11.82		
	>20,000	60(18.2)	48.78 ± 10.80		
Residence status	City	319(96.7)	47.52 ± 11.86	2.48	0.117
	Rural	11(3.3)	41.73 ± 15.88		
Marital status of parents	Normal marriage	319(96.7)	47.35 ± 11.83	0.05	0.827
	Divorce or separation	11(3.3)	46.55 ± 17.48		
Mainly caregivers	Parents	113(34.2)	47.36 ± 11.78	0.20	0.900
	Parents and Grandparents	64(19.4)	47.14 ± 13.80		
	Grandparents	138(41.8)	47.13 ± 11.53		
	Other	15(4.5)	49.60 ± 11.19		

### Means, standard deviations, and association of coparenting, parental stimulation and children’s social–emotional ability

Table 2 provides the mean, SD, and correlation of the relevant variables. The mean score of coparenting was 62.49 (SD = 13.27). The mean scores of parental stimulation, mother’s and father’s stimulation were 11.61 (SD = 4.45), 10.26 (SD = 5.02), and 5.57(SD = 4.70), respectively. The mean score of social emotion was 47.32 (SD = 12.03).

**Table 2:** Means, standard deviations, and correlations of coparenting, parental stimulation and children’s social emotional ability (*N* = 330).

Variables	1	2	3	4	5
1. Coparenting	1				
2. Parental stimulation	0.263**	1			
3. Mother’s stimulation	0.267**	0.818**	1		
4. Father’s stimulation	0.363**	0.444**	0.493**	1	
5. Social emotional ability	0.165**	0.261**	0.208**	0.169**	1
*M*	62.49	11.61	10.26	5.57	47.32
SD	13.27	4.45	5.02	4.70	12.03

M, mean; SD, standard deviation. *P < 0.05, **P < 0.01.

In coparenting, the highest dimension of the mean score was mutual damage, and the lowest dimension was housework distribution. In maternal stimulation, the items with high scores were playing games with toys and singing nursery rhymes, while the items with low scores were telling stories. Among paternal stimulation, the items that scored high were playing games with toys, while the items with low scores were telling stories and singing nursery rhymes. In terms of social emotion, 10.3% of children had abnormal social emotion, the dimension with the highest score was imitation or play, and the dimension with the lowest score was compliance. The study variables pairwise showed a positive correlation.

### Factors associated with social–emotional ability

Hierarchical regression analysis was employed to explore factors associated with social–emotional ability (Table 3). Model 1 included socio-demographic characteristics with statistical difference in univariate analysis (child’s age, family monthly income, feeding pattern, delivery way, low birth weight), coparenting entered Model 2 and parental stimulation entered Model 3. The *R^2^* of Model 3 is 0.317 and the adjusted *R^2^* is 0.293 (*F* = 13.408, *p* < 0.01). The factors that associated with children’s social–emotional ability are as follows: child age, family monthly income, feeding pattern within 6 months of age, coparenting score and parental stimulation. Coparenting explained 2.1% of the variance in social–emotional ability. Coparenting and parental stimulation accounted for 7.4% of the variance.

**Table 3:** Multi-stratified regression analysis of social emotional ability (*N* = 330).

Variables	Model 1			Model 2			Model 3		
	*B*	*β*	*t*	*B*	*β*	*t*	*B*	*β*	*t*
Child’s age	8.252	0.332	6.668**	8.827	0.355	7.148**	9.558	0.385	7.966**
Monthly family income (RMB)									
7,000–10,000	3.856	0.148	2.277*	3.681	0.141	2.201*	4.184	0.161	2.590*
10,000-20,000	5.710	0.220	3.353**	5.234	0.202	3.102**	4.326	0.167	2.643**
>20,000	5.119	0.164	2.655**	4.889	0.157	2.568*	4.723	0.152	2.573*
Feeding method									
Artificial feeding	−5.777	−0.160	−3.017**	−5.653	−0.157	−2.991**	−4.238	−0.118	−2.298*
Mixed feeding	0.051	0.002	0.038	0.558	0.021	0.423	1.313	0.050	1.024
Delivery way	−1.534	−0.064	−1.254	−1.608	−0.067	−1.332	−1.494	−0.062	−1.282
Low birth weight	4.600	0.077	1.562	4.815	0.081	1.656	5.140	0.086	1.833
Coparenting				0.145	0.160	3.114**	0.096	0.106	2.093*
Parental stimulation							0.676	0.250	5.002**
*F*	11.272**			11.390**			13.408**		
*R^2^*	0.241			0.263			0.317		
Adjust *R^2^*	0.219			0.240			0.293		

B, unstandardized coefficients; β, standardized coefficients; *t*, *t*-value. *P < 0.05, **P < 0.01.

### Mediation analyses

**Figure 1:**
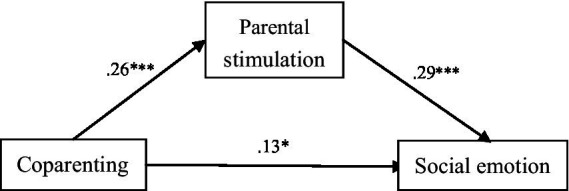
Path analysis of coparenting, parental stimulation and social emotion, with standardized beta weights and significant level (*P < 0.05, **P < 0.01, ***P < 0.001).

**Figure 2:**
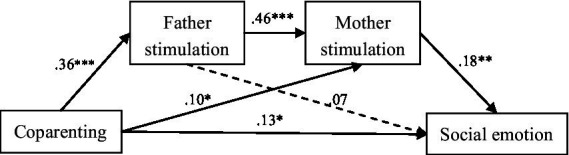
Path analysis of coparenting, father stimulation, mother stimulation and social emotion, with standardized beta weights and significant level (*P < 0.05, **P < 0.01, ***P < 0.001).

As shown in Figures 1, 2, coparenting was related to 3 mediators (parental stimulation, paternal stimulation, and maternal stimulation), which in turn parental stimulation and maternal stimulation were significantly related to social–emotional ability. Figures 1, 2 demonstrated a very good fit, respectively (Hu and Bentler, 1999): CMIN/DF = 3.003/2.333; GFI = 0.962/0.964; AGFI = 0.920/0.930; CFI = 0.9680/0.973; RMSEA = 0.078/0.064; SRMR = 0.0437/0.040.

Tables 4, 5 shown the direct and indirect effects of coparenting on social–emotional ability. In Table 4, parental stimulation mediated the association between coparenting and social–emotional ability (*β* = 0.011, bootstrap 95% *CI* = 0.006, 0.018). In Table 5, maternal stimulation independently mediated 10% of the association between coparenting and social–emotional ability (*β* = 0.003, bootstrap 95% *CI* = 0.001, 0.007). Additionally, the father’s stimulation and the mother’s independently mediated 13.3% of the association between coparenting and social–emotional ability (*β* = 0.004, bootstrap 95% *CI* = 0.002, 0.008). These results indicate that the positive association between coparenting and social–emotional ability was modestly explained by higher levels of parental stimulation.

**Table 4:** Direct, indirect, and total effects of coparenting and parental stimulation on social emotion.

Relationships	Point estimates	Boot SE	Bias-corrected 95% *CI*	
			Lower	Upper
Total effects	0.030	0.010	0.011	0.050
Direct effects	0.019	0.009	0.002	0.038
Indirect effects	0.011	0.003	0.006	0.018

**Table 5:** Direct, indirect, and total effects of coparenting, father stimulation, mother stimulation and parental stimulation on social emotion.

Relationships	Point estimates	Boot SE	Bias-corrected 95% *CI*	
			Lower	Upper
Total effects	0.030	0.010	0.015	0.048
Direct effects	0.038	0.010	0.004	0.038
Total indirect effects	0.011	0.004	0.005	0.018
Indirect effects 1	0.004	0.002	0.002	0.008
Indirect effects 2	0.004	0.003	−0.002	0.009
Indirect effects 3	0.003	0.002	0.001	0.007

Indirect effects 1: Coparenting to Father stimulation to Mather stimulation to Social emotion.

Indirect effects 2: Coparenting to Father stimulation to Social emotion.

Indirect effects 3: Coparenting to Mather stimulation to Social emotion.

## Discussion

Our study investigated the status of coparenting, parental stimulation, and social–emotional ability of children aged 12 to 36 months in the context of Chinese culture, and analyzed the relationship among coparenting, parental stimulation, and social–emotional ability. We found that the mother’s stimulation was at the middle-upper level, the father’s stimulation was at the middle-lower level, and 10.3% of children had abnormal social emotion. Parental stimulation partially mediates the relationship between coparenting and social–emotional ability. Our research highlights the importance of coparenting and parental stimulation for improving children’s social–emotional ability.

The parental stimulation score in our study is lower than that in the United States on the positive parenting behavior of 1–5-year-old families (Cprek et al., 2015), which may be because children are mostly taken care of by grandparents, and parents do not spend enough time with their children in the context of Chinese culture. Storytelling scored the lowest, and playing games with toys scored the highest among interactive games. Previous studies have shown (Yue et al., 2019) that when caregivers engaged with children in telling stories, children scored higher on the Mental Development Index and Psychomotor Development Index scores by 7.62 and 3.15, respectively. This suggests that caregivers need to balance the types of interactions with their children, and they need to pay more attention to interactions with children such as reading and storytelling in the future.

In our study, the father’s stimulation score is lower than the mother’s stimulation score, which is consistent with the research results of Cuartas et al. (2020). This may be due to the traditional concept that women are mostly family caregivers and men are mostly responsible for family economics which leads to a lower frequency of father’s involvement in the interaction (Fikree and Pasha, 2004). Previous studies have shown (Jeong et al., 2016) that the child’s early development score is reduced by 0.19 points when the father and the child without interactive stimulation compared with a high level of interactive stimulation. This suggests the importance of father stimulation. Although fathers need to take more responsibility for raising the family, fathers still need to take time to interact with their children to promote their development.

Regression analysis results showed that coparenting was positively correlated with children’s social–emotional ability (*B* = 0.096, *p* < 0.05), and the higher the coparenting score, the higher the children’s social–emotional ability. A previous meta-analysis also showed (Teubert and Pinquart, 2010) that coparenting is related to children’s internal behaviors (such as anxiety, depression, withdrawal, etc.), external symptoms (hyperactivity, antisocial, aggressive, etc.), and parent–child attachment. This may be because parents who support each other in coparenting are able to show more responsiveness to their children, thus promoting the positive development of children (Zemp et al., 2018). This suggests that in the process of raising children, parents need to support and cooperate, and parents need to actively participate in raising children to fully stimulate their children’s development potential.

Regression analysis results showed that parental stimulation was positively correlated with children’s social–emotional ability (*B* = 0.676, *p* < 0.01). The higher the parental stimulation score, the higher the children’s social–emotional ability score. Our results are consistent with previous research results (Jeong et al., 2020; Scherer et al., 2019), such as a study in Pakistan showed that mothers’ responsive interactions with their children can affect children’s social–emotional developmental outcomes (Scherer et al., 2019). One to three years old is the fastest period for children’s brain development, and various types of parent–child interaction and stimulation are conducive to the rapid growth of the connections between children’s neurons (Fox et al., 2010). This prompts parents, families, and society to pay attention to and improve stimulation: parents can improve their parenting knowledge and skills, and spend time participating in children’s interaction; the family level can improve family parenting resources, such as enriching books and toys at home; the social level can establish a complete social support system for parents, such as providing parents with scientific parenting guidance and policy support.

The mediation results showed that coparenting was associated with child social–emotional abilities and that parental and maternal stimulation partly explained this association, which is consistent with the coparenting ecological model. The path from coparenting to father’s stimulation to mother’s stimulation to children’s social–emotional ability was significant. However, the path from coparenting to father’s stimulation to children’s social–emotional ability was non-significant. Importantly, the indirect effect size of the chain mediation was small in this study, which may be related to the cross-sectional study design and the interaction between paternal and maternal stimulation. In the future, longitudinal studies and the inclusion of other variables (e.g., children’s temperament and quality of marriage) can be designed for further exploration.

Paternal stimulation and maternal stimulation play a chain mediating role in the relationship between coparenting and social–emotional ability. This shows that good cooperation and support between fathers and mothers in parenting was associated with fathers’ active participation in parenting practices and interactions with children. The research result of Liu et al. (2016) also showed that the quality of coparenting can affect the father’s parenting practice. In addition, we found the stimulating interaction between fathers and children was also related to the stimulating interaction between mothers and children, which is consistent with the research results of Lee et al. (2020) which showed that father’s participation in caring for children can reduce mother’s stress and increase mother’s sensitive parenting behavior. Therefore, good coparenting was associated with the father’s active participation in children’s stimulation interaction, and father’s high-quality stimulation was related to mother’s high-quality stimulation so that children’s social–emotional ability can be fully developed. The importance of coparenting and parental stimulation has also been confirmed by a previous study in Vietnam which showed that intervention in coparenting and father-child interaction can improve the parent–child relationship, the child’s language and social–emotional development outcomes (Rempel et al., 2017).

In addition, the mediating effect of paternal stimulation on coparenting and children’s social–emotional ability is not significant, which may be because the path model also includes mother’s stimulation. When mother’s stimulation is high, father’s stimulation has a smaller association with children’s social–emotional ability. The study of Jeong et al. (2016) showed that when the mother’s sensitive interaction was low, the father’s sensitive interaction had a more significant correlation with the scores of children in early childhood. This suggests that not only the mother’s stimulation should be improved, but also the father’s stimulation. According to previous research results, when the mother’s stimulation interaction is at high risk, the father’s high-quality stimulation can also buffer the adverse impact of the mother’s low level of stimulation on the child’s developmental outcome (Jeong et al., 2016).

### Limitation

The cross-sectional nature of this study has limitations in determining the causal relationship between coparenting, parental stimulation, and social–emotional ability. Longitudinal research can be carried out in the future to explore the relationship between the three variables. The convenience sampling method was adopted in this study, and the representativeness of the population is limited to a certain extent. Future research can adopt random sampling method and carry out multi-center and large-sample research. The indirect effect size of this study was relatively small, and the explanatory power was not high enough. Children’s social emotions are also affected by other factors, such as children’s temperament, parents’ parenting beliefs, etc. Future research can include more variables to explore their impact on children’s social–emotional ability. In the measurement of parental stimulation and social–emotional competence, the use of parental reports may cause reporting bias. Future research can explore the use of observational measurement tools. In our study, the coparenting quality and father’s stimulation were reported by the mother, which may cause bias. Future research can include both mothers and fathers to ensure the accuracy of the measurement.

## Conclusion

We found that paternal stimulation and maternal stimulation played a chain mediating role between coparenting and children’s social–emotional ability. The results of our study emphasize the importance of coparenting and parental stimulation on the development of children’s social–emotional ability, which suggests that in the process of parenting children, parents need to support and cooperate, and fathers need to actively participate in parenting. However, the indirect effect of parental stimulation accounted for 36.7% of the total effect, and the explanatory power is not high enough. In the future, we can explore the mediating role played by parents’ parenting pressure, parenting competence, marital quality, and family parenting environment. Our study provides the basis for future interventions to enhance children’s social–emotional development by directing and enhancing coparenting and parental stimulation.

## Data Availability

The raw data supporting the conclusions of this article will be made available by the authors, without undue reservation.
